# An internet-based algorithm for diabetic foot infection during the COVID-19 pandemic

**DOI:** 10.1186/s13047-020-00405-z

**Published:** 2020-06-17

**Authors:** Chao Liu, Wen-Li Shi, Jia-Xing You, Hong-Ye Li, Lin Li

**Affiliations:** 1 Department of Orthopedics, Zhejiang University School of Medicine Sir Run Run Shaw Hospital, #3 E. Qingchun Rd, Hangzhou, 310016 China; 2 Department of Endocrinology, Zhejiang University School of Medicine Sir Run Run Shaw Hospital, #3 E. Qingchun Rd, Hangzhou, 310016 China

The fallout from the coronavirus disease 2019 (COVID-19) is not just limited to the infected individuals and, moreover, has significant influences on the patients with chronic diseases. It is estimated that 463 million adults have diabetes worldwide, and diabetic foot ulcer (DFU) is one of the most serious complications of diabetes, with a prevalence of 6.3% [1, 2]. Most patients with DFU need to regularly visit healthcare providers to receive adequate wound care and obtain the maintenance medications for diabetes. In the current crisis, however, there is a fear of exposure in public environments because of the possibility of respiratory droplet-borne rapid virus spread. Furthermore, mass quarantines and restrictions on public transport have inevitably become a major barrier to the traditional face-to-face consultations for patients with diabetic foot infection (DFI), leading to serious consequences, such as the increases of the risks of lower extremity amputation and mortality. Thus, an alternative modality for those patients during the COVID-19 pandemic is urgently needed [3]. Herein, we, members of a multidisciplinary team (MDT), share our first-hand experience at Sir Run Run Shaw Hospital of Zhejiang University, which is one of the first medical services to implement Internet-based healthcare constructions in China. Our multidisciplinary team was formed in 2016, consisting of diabetes specialists, orthopedists, vascular surgeons, physicians specializing in infectious diseases, diabetes specialist nurses, and foot care and screening nurses, coordinated by an attending diabetes specialist and orthopedist. Herein, we report an unvalidated system being developed quickly in response to an urgent public health need. In this time of health crisis, healthcare practitioners are seeking the best models of care. Although this is not a scientific-based study, it would benefit if there is supporting evidence on the algorithm and more detail on the treatment pathways would be beneficial.

Building on the popularization of the Internet and smartphones, we began providing online consultation services for patients with a new DFU episode via instant messaging (e.g. Ngari Doctors, WeChat, etc), networking platform and remote consultation system (Fig. 1). All patients should be first screened for the COVID-19 infection if they have respiratory syndrome and/or history of close contact with confirmed cases. After the demographic data are collected, patients are instructed to upload appropriate photographs and short videos of their wounds to the networking platform for further evaluation. The patients were instructed to meet the following photo or video requirements (audited by our MDT online): a dark color background for contrast, standard room lighting, whole foot/ulcer, and multiple angles. Written informed consent was obtained from all the participants. Based on the original informed consent, we added the informed consent for invasive examination and treatment during the COVID-19 outbreak. We stored all the files in our hospital medical record system. All diabetic foot complications were properly treated, but especially during the COVID-19 outbreak, when limb ischemia, loss of protective sensation, and Charcot neuroarthropathy caused moderate to severe infection, they were treated at the hospital promptly. If these complications were not co-infections, patients were followed up and observed at home, waiting for the end of the COVID-19. Based on the International Working Group on the Diabetic Foot classification [4, 5], specifically using the International Working Group on the Diabetic Foot/Infectious Diseases Society of America (IWGDF/IDSA) system, we stratify patients by the severity of the DFU infection and formulate recommendations for each group as follows (Fig. 2). Although the IWGDF/IDSA system for infection classification may not be able to evaluate the disease completely and accurately, this evaluation system is relatively simple and reliable during the COVID-19 outbreak. Patients and their families or community general practitioners were roughly graded based on the appearance of the wounds, body temperature, heart rate, and breathing rate. Furthermore, they were instructed to upload appropriate photographs and short videos of their wounds to the networking platform for further evaluation. For patients who were not technologically savvy, families and community workers were urged to help them, especially since the COVID-19 outbreak coincided with the Chinese New Year, and families tended to stay at home.

**Fig. 1:**
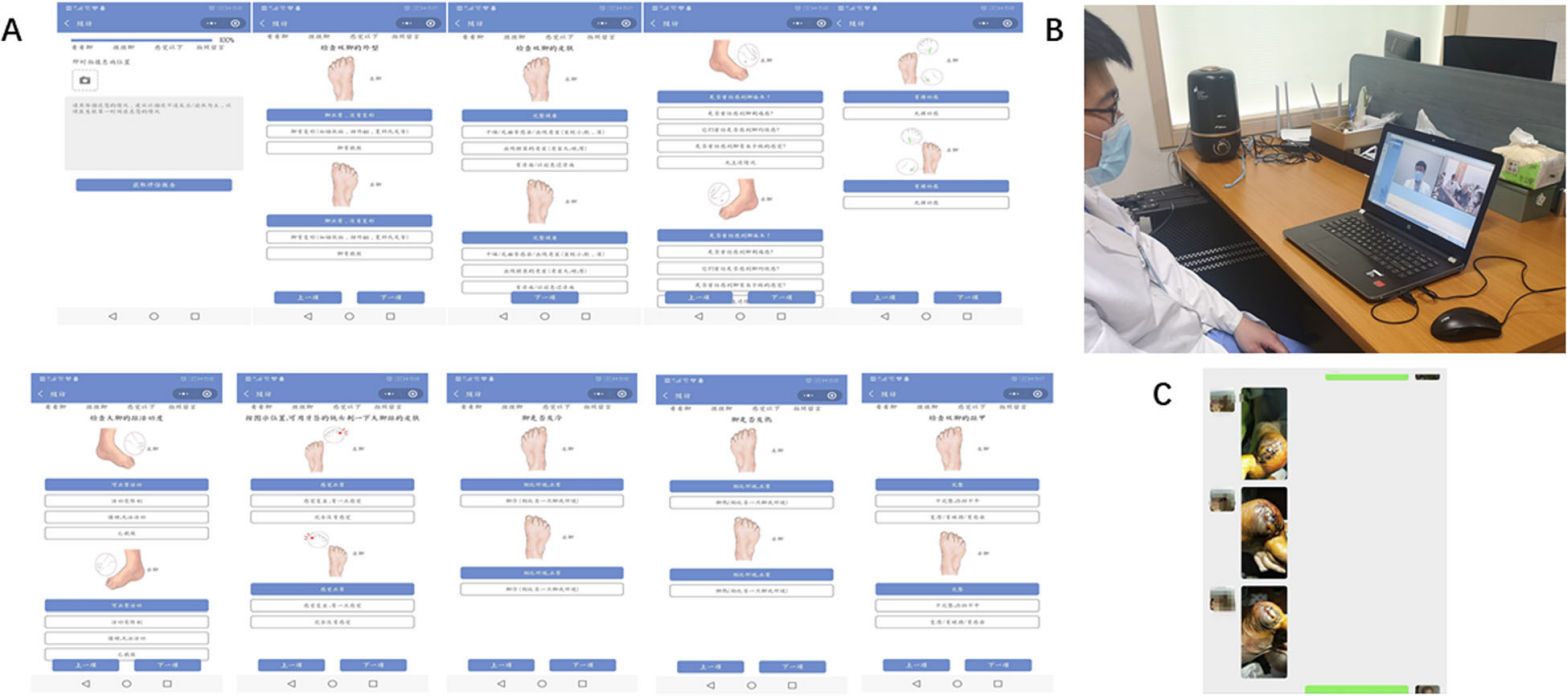
We provide online consultation services for patients with DFU via networking platform (**a**): Patients use this APP to upload photos of the foot according to the set process, and follow the prompts to inform the appearance of the foot, skin, motor ability, pain, numbness and toes; remote consultation system (**b**): Local medical staff can establish real-time contact with our team through the remote consultation mode to discuss the disease and determine the further diagnosis and treatment plan; and instant messaging (e.g. Ngari Doctors, WeChat, etc) (**c**): Send foot photos and videos using instant messaging tools that are widely used in China

**Fig. 2:**
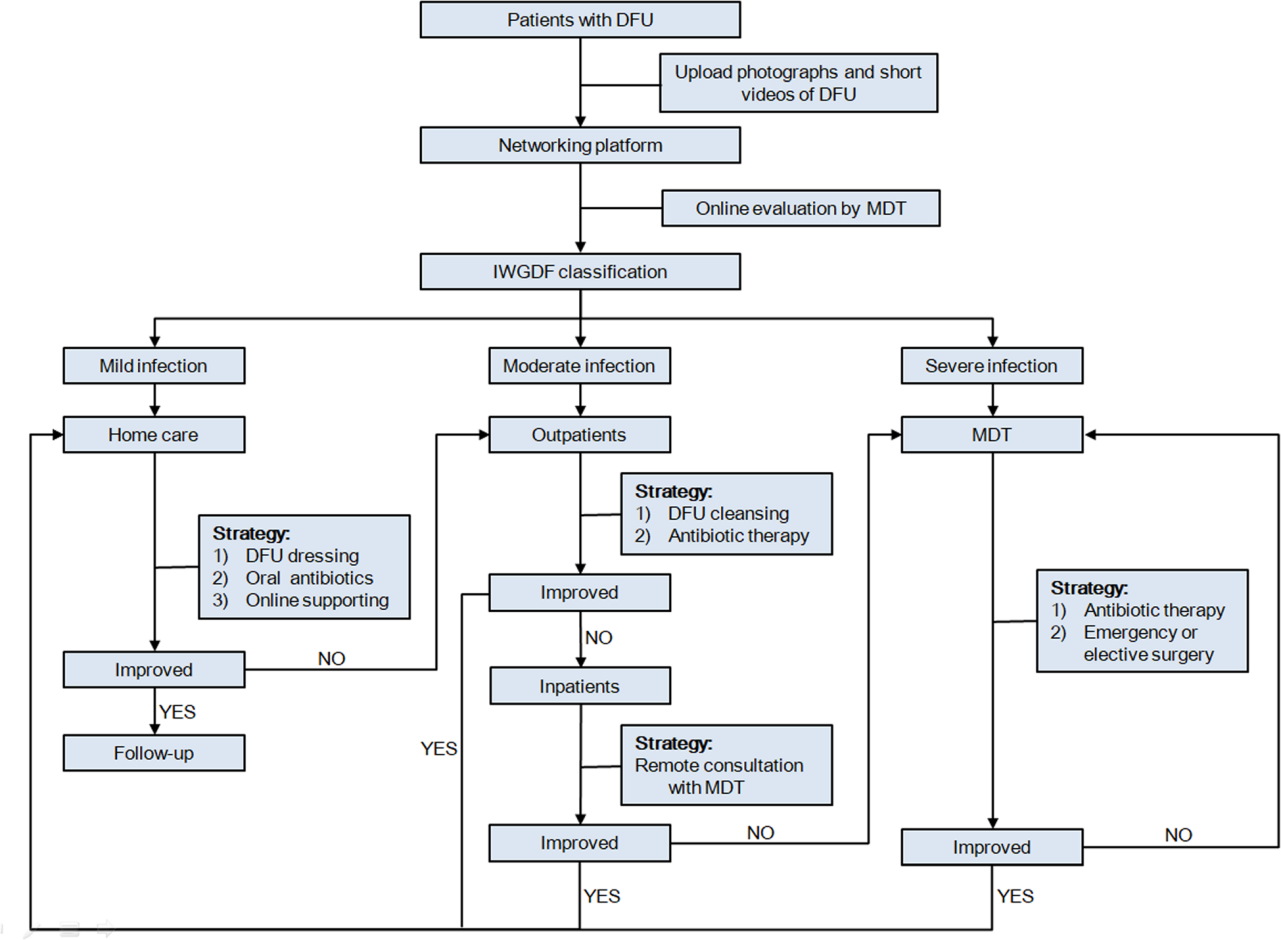
Flow chart for treatment of diabetic foot ulcer during the coronavirus disease 2019 outbreak. DFU = diabetic foot ulcer. MDT = multidisciplinary team. IWGDF = International Working Group on the Diabetic Foot

For patients with mild infection, we recommend daily DFU dressing by themselves or relatives to be carried out under online guidance and supervision. House call practice by community general practitioners is available in elderly or empty-nest patients (ie. without children, or whose children left home and worked elsewhere). Oral short-duration (1–2 weeks) antibiotic therapy usually suffices to fight infection. After approval by the Healthcare Security Administration of Zhejiang province, China, dressing products and maintenance medications are shipped by express delivery [6]. Moreover, online education, glucose monitoring and mental health supporting are also available. Notably, photographs of the DFU should be updated at every dressing change, to facilitate the modulation of the home-care strategy in compliance with the wound status.

For patients with moderate infection, we recommend wound cleansing at nearest outpatients in combination with antibiotic therapy, which is initially administered parenterally and then switched to oral within a week. While most patients in this group do not need hospitalization, some certainly should be, especially those have limb-threatening DFI and osteomyelitis. Through the remote consultation system, the therapeutic strategy, such as debridement and amputation, is audited by our MDT. To minimize the potential risks of nosocomial COVID-19 infection, once there are early signs of DFI improvement, patients are discharged to receive home care similarly as patients with mild infection. Offloading methods include surgical shoes, football dressing, boots, and total contact cast. Debridement can be undertaken using physical (e.g., surgical, sharp, or hydro-debridement), biological (larvae), autolytic (hydrogels), or biochemical (enzymes) methods. Moreover, sharp debridement is an essential part of wound care. If the DFU worsens between consultations, the patient will be referred and transported to our MDT promptly.

Patients with severe infection, usually accompanies by systemic inflammatory responses, are referred in a timely manner to our multidisciplinary team. A chest computer tomography and nucleic acid detection for coronavirus are mandatory to distinguish from the COVID-19 infection. Antibiotic therapy is first managed empirically and then modified according to the results of an antibiogram following culture. Emergency operation is usually required for patients with life-threatening DFI. Because of the time-lapse between urgent interventions and a laboratory-confirmed diagnosis of COVID-19, aggressive protective measures, such as N95 masks, goggles, and a negative pressure operating room, are imperative to ensure the safety of medical personnel. Besides, peripheral nerve block outweigh general anesthesia in protecting anesthetists from occupational exposure to infections of respiratory tract. Cases of confirmed COVID-19 should receive wound care as well as postoperative pneumonia treatment in the isolation ward under the guidance of the National Health Commission of China [7]. Otherwise, standard clinical protocols are to be followed, and patients would be transferred to the integrated healthcare service or discharge to home care when medically stable.deb.

An Internet-based algorithm for patients with DFI during the COVID-19 outbreak has been established, this algorithm might help political leaders and health authorities to allocate and optimize medical resources. We hope that some of our experiences could assist others in managing DFU infection in their regions. Besides the infection, other critical factors such as acute or chronic critical limb ischemia, loss of protective sensation and charcots neuroarthropathy also need to be taken into consideration. As the diabetic foot treatment results will take time to follow up, the outcomes are still being worked on and will be available in the future.

## Data Availability

Not applicable.

## Abbreviations

COVID-19: The coronavirus disease 2019
DFU: Diabetic foot ulcer
DFI: Diabetic foot infection
MDT: Multidisciplinary team
